# Serum neurofilament light chain in pediatric spinal muscular atrophy patients and healthy children

**DOI:** 10.1002/acn3.51449

**Published:** 2021-09-04

**Authors:** Elisa Nitz, Martin Smitka, Jens Schallner, Katja Akgün, Tjalf Ziemssen, Maja von der Hagen, Victoria Tüngler

**Affiliations:** ^1^ Department of Neuropediatrics Medizinische Fakultät Technische Universität Dresden Dresden Germany; ^2^ Department of Neurology Center of Clinical Neuroscience Universitätsklinikum Carl Gustav Carus Technische Universität Dresden Dresden Germany; ^3^ University Center for Rare Diseases Medizinische Fakultät Carl Gustav Carus Technische Universität Dresden Dresden Germany

## Abstract

**Objective:**

The aim of this study was to evaluate neurofilament light chain as blood biomarker for disease activity in children and adolescents with different types of spinal muscular atrophy (SMA) and establish pediatric reference values.

**Methods:**

We measured neurofilament light chain levels in serum (sNfL) and cerebral spinal fluid (cNfL) of 18 children with SMA and varying numbers of *SMN2* copies receiving nusinersen by single‐molecule array (SiMoA) assay and analyzed correlations with baseline characteristics and motor development. Additionally, we examined sNfL in 97 neurologically healthy children.

**Results:**

Median sNfL levels in treatment‐naïve SMA patients with 2 *SMN2* copies are higher than in those with >2 *SMN2* copies (*P* < 0.001) as well as age‐matched controls (*P* = 0.010) and decline during treatment. The median sNfL concentration of healthy controls is 4.73 pg/mL with no differences in sex (*P* = 0.486) but age (*P* < 0.001). In all children with SMA, sNfL levels correlate strongly with cNfL levels (*r* = 0.7, *P* < 0.001). In children with SMA and 2 *SMN2* copies, sNfL values correlate with motor function (*r* = –0.6, *P* = 0.134), in contrast to older SMA children with >2 *SMN2* copies (*r* = –0.1, *P* = 0.744).

**Interpretation:**

Reference sNfL values of our large pediatric control cohort may be applied for future studies. Strong correlations between sNfL and cNfL together with motor function suggest that sNfL may be a suitable biomarker for disease activity in children with 2 *SMN2* copies and those with >2 *SMN2* copies within their initial stages during early childhood.

## Introduction

As the most common genetic cause of infant mortality, spinal muscular atrophy (SMA) results from deleterious variants in *SMN1*, which lead to deficiency of survival motor neuron protein (SMN). This deficiency of SMN is followed by degeneration of motor neurons and associated with progressive atrophy of skeletal and respiratory muscles.^1^ An *SMN1* paralog, *SMN2*, is considered the most important phenotype modifier in a copy‐dependent manner and partially compensates the deficit of *SMN1*.^2^

By convention, childhood SMA is classified into subtypes based on age at onset and maximal acquired motor skills: severe type I (SMA I, MIM 253300), intermediate type II (SMA II, MIM 253550), and mild type III (SMA III MIM 253400).^1^ In type I, the majority of individuals carry 2 *SMN2* copies and if left untreated need permanent ventilation or die from respiratory failure within the first two years of life.^3^ Most SMA II patients carry 3 *SMN2* copies and may sit, yet are unable to stand or walk. In SMA III, most individuals carry 4 *SMN2* copies and are able to walk, but require wheelchair assistance later in life.^4^

The anti‐sense oligonucleotide nusinersen (Spinraza, Biogen, Cambridge, USA) has been approved as the first of three disease‐modifying therapeutic agents and is given intrathecally in periodic intervals.^5^, ^6^ Treatment options expanded with approval of onasemnogene abeparvovec, an adeno‐associated virus 9‐based gene delivery, and risdiplam, an orally deliverable small molecule splicing modifier.^7^ While nusinersen has been the first drug that demonstrates clinical efficacy, the extent and progress rate of motor function improvements under treatment have been described as diverse.^5^, ^8^ Therefore, objectively measured molecular biomarkers, which reflect pathologic processes and their response to therapeutic interventions, may be a good addition to current diagnostic means. Neurofilaments have gained increasing attention in this regard.^9^, ^10^, ^11^ As structural constituents of the axoskeleton, neurofilaments are neuron‐specific and composed of four subunits including neurofilament light chain (NfL) and heavy chain (NfH).^12^ Following neuroaxonal damage, neurofilaments are released into the interstitial fluid, the cerebral spinal fluid (CSF) and peripheral blood. Despite significantly lower blood concentrations, reproducible measurements were made feasible by ultra‐sensitive single‐molecule array (SiMoA) assays that are able to detect serum proteins at subfemtomolar concentrations.^13^, ^14^

Biomarkers that are conveniently quantifiable in the blood are desirable. To date, few studies aimed at addressing neurofilament as a potential blood biomarker for SMA. One study measured NfH plasma concentrations in children with SMA I by means of a microfluidic enzyme‐linked lectin immunoassay (Simple Plex platform),^9^ another evaluated serum NfL (sNfL) in adolescent and adult patients with SMA II–III using SiMoA,^11^ with discrepant results suggesting potential age dependency. So far, there has been no information concerning the value of neurofilament as a biomarker of disease activity in children with later‐onset SMA. In order to assess the value of NfL as a diagnostic and monitoring biomarker in SMA and evaluate whether elevated NfL levels are associated with SMA subtype or age, we quantified NfL concentrations in serum and CSF of children and adolescents with SMA carrying either 2 *SMN2* copies or >2 *SMN2* copies before and during therapy with nusinersen over a period of up to 34 months. Owing to the reliably reproducible SiMoA method, the data of our large and broad age‐ranged pediatric control cohort may be applied as reference values for further studies investigating sNfL in SMA and other pediatric neurodegenerative diseases.

## Methods

### Standard protocol approvals, registrations, and patient consents

The study was approved by the Ethics Committee of the Technische Universität Dresden, Germany (183042019) with written informed consent obtained from each participant and/or their legal representative and conducted in accordance with the International Conference on Harmonization guidelines for Good Clinical Practice and the World Medical Association Declaration of Helsinki.

### SMA patient assessment

In total 18 SMA patients ranging from 18 days to 17.2 years of age (at time of inclusion) were included in this study. A distinction was made between SMA patients with 2 *SMN2* copies, that are most likely classified as SMA I, and SMA patients with a later‐onset type of disease and >2 *SMN2* copies, most likely classified as SMA II–III. Further information on the study participants is detailed in the results section in Table 1. Baseline and serial serum and CSF samples along with clinical information were obtained on a regular, nusinersen‐synchronized basis, prior to intrathecal drug administration. The recommended dosing regimen was followed and included a baseline, prior to the initiation of treatment (dose 1: month 0); an initial loading phase (dose 2: month 0.5, dose 3: month 1, and dose 4: month 2) as well as a maintenance phase with repeated intervals of 4 months (dose 5: month 6, dose 6: month 10, dose 7: month 14, dose 8: month 18, dose 9: month 22, dose 10: month 26, dose 11: month 30 and dose 12: month 34). Treatment duration ranged from 2 to 34 months (median of 22 months) with the number of samples per time point detailed in Supplementary Table S1. SMA patients were compared to age‐matched controls without SMA from our reference cohort. Motor function was measured using the Children's Hospital of Philadelphia Infant Test of Neuromuscular Disorders (CHOP INTEND, point scale of 0–64) for patients younger than 2 years and the Hammersmith Functional Motor Scale Expanded (HFMSE, point scale 0–66) for patients older than 2 years. In both tests, higher scores indicate better motor function.^15^, ^16^

**Table 1:** Characteristics of treatment‐naïve SMA patients.

Patient (#)	*SMN2* copy number	SMA subtype	Sex	Age at baseline (years)	cNfL baseline (pg/mL)	sNfL baseline (pg/mL)	Number of doses received	Number of age‐matched controls
1	2	I	m	0.05	23800	676	4	6
2	2	I	f	0.22	6590	381	10	6
3	2	I	m	0.25	19000	1030	4	6
4	2	I	f	0.93	4020	335	4	6
5	>2 (3)	II	m	1.05	489	34.50	6	12
6	>2 (3)	II	f	3.44	259	22.90	12	4
7	>2 (3)	II–III	f	3.61	157	11.70	9	4
8	>2 (3)	II	f	4.29	246	n.a.	10	8
9	>2 (3)	II–III	f	4.43	381	23.60	12	8
10	>2 (4)	II–III	m	7.90	165	6.55	10	3
11	>2 (4)	II–III	m	7.90	141	7.72	10	3
12	>2 (3)	II	f	8.99	314	10.42	9	1
13	>2 (3)	II	m	9.84	244	5.35	12	4
14	>2 (3)	II	m	10.72	106	5.24	8	5
15	>2 (3)	II	m	11.07	112	10.80	10	6
16	>2 (3)	IIIa	f	15.18	485	10.70	6	6
17	>2 (3)	IIIa	f	15.43	169	10.10	7	6
18	>2 (3)	III	f	17.21	207	4.29	5	3

Demographics of SMA patients and their age‐matched controls. Indicated are patient labels, count of *SMN2* copies, most likely classified SMA subtype, sex and age at the initiation of treatment. Additionally, baseline sNfL and cNfL levels, the total number of nusinersen doses received until data cut as well as the number of age‐matched healthy controls are given. n.a., data not available; SMA, spinal muscular atrophy; cNfL, CSF neurofilament light chain; sNfL, serum neurofilament light chain.

### Controls

In consideration of the lack of reference values in childhood^12^ one‐time serum samples from 97 healthy children (aged 2.4 months–18.1 years), who met inclusion criteria (exclusion of existing neurodegenerative, recent acute or chronic inflammatory disease, systemic immune diseases, drugs affecting the immune or nervous system, injuries to the CNS in the last year) were collected. The cohort included age‐matched controls for all SMA patients.

### CSF and serum analysis

CSF, obtained via lumbar puncture and serum from peripheral blood was taken, centrifuged and stored at −80°C. NfL concentrations in CSF and serum were measured by single‐molecule array (SiMoA) assay performed on the instrument HD‐1 Analyzer (Quanterix, Lexington, MA, USA)^13^, ^17^ using the two‐step Assay Dilution 2.0 protocol for the NF‐light Advantage kit from Quanterix, which employs an anti‐NfL monoclonal antibody and calibrators produced by UmanDiagnostics (Umeå, Sweden). Analyses were performed in duplicates. Interassay coefficients of variation (CV) were less than 10%. SiMoA, which confine enzyme labels within femtoliter volumes and thereby enable the detection of single molecules, are currently considered the most sensitive method for the analysis of NfL in blood.^14^

### Statistical analysis

According to the D'Agostino‐Pearson test, data were not normally distributed. Therefore, nonparametric tests were performed. Reference values by groups of sex and age as well as baseline characteristics of patients with 2 and >2 *SMN2* copies were pairwise analyzed by Mann–Whitney‐U and Kruskal–Wallis tests. Changes in parameter values (NfL and motor function) over the course of time were evaluated by Wilcoxon matched‐pairs signed‐rank tests and indicated by descriptive statistics. Associations between NfL in CSF (cNfL) and serum (sNfL) as well as between sNfL concentrations and motor function scores were assessed with Spearman and Pearson correlation coefficients *r* and linear regression. All analyses and plots were implemented using GraphPad Prism version 9 (GraphPad Software Inc., San Diego, California). The tests were two‐sided and *P* values along with their 95% confidence interval (CI) were presented. Results with *P* values ≤ 0.05 were interpreted as statistically significant.

## Results

### Reference serum NfL values in children

**Figure 1:**
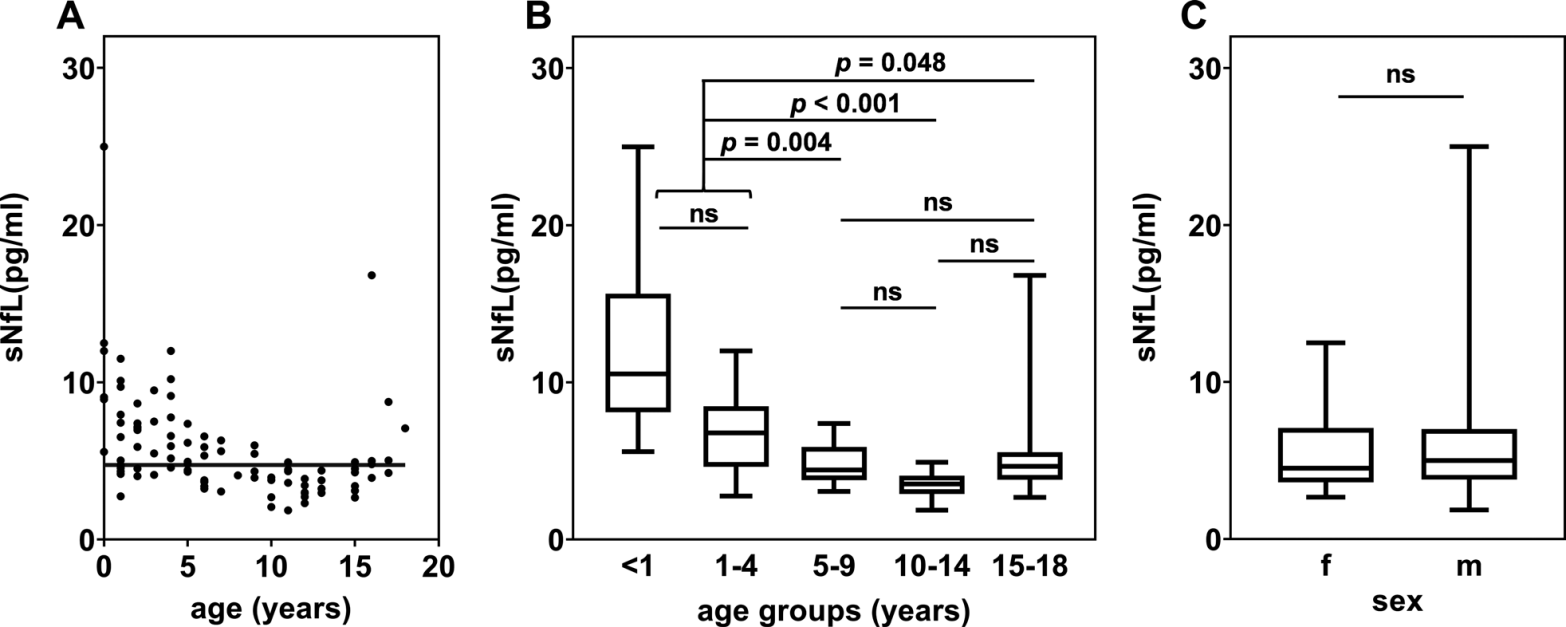
sNfL in pg/mL in neurologically healthy children (*n* = 97). (A) Dot plot association between sNfL and age (in years) with the median sNfL value of 4.73 pg/mL represented by a horizontal line. (B, C) Boxplots of sNfL levels in healthy controls separated by age groups (<1, 1–4, 5–9, 10–14, 15–18 years) and sex (female vs. male). Given are median, minimum, maximum levels and the interquartile range (25% and 75% percentile) as well as the *P* value showing significant differences (ns, non‐significant) between groups (Mann–Whitney‐U and Kruskal–Wallis test).

To explore age and sex related sNfL value changes, we assessed sNfL values from 97 individuals aged 2 months to 18 years (median age of 6.3 years) without any neurodegenerative or inflammatory disorder (overall median of 4.73 pg/mL, range: 1.84–25.00 pg/mL, *n* = 97; Fig. 1A). Comparison of different age groups revealed significantly higher median sNfL levels in infants and children until the age of 4 years (7.12 pg/mL, range: 2.73–25.00 pg/mL, *n* = 38) compared to children and adolescents aged between 5 and 18 years (4.07 pg/mL, range: 1.84–16.80 pg/mL, *n* = 59, 95% CI [1.790, 3.750], *P* < 0.001; Fig. 1B). Median value comparison of 43 female children (4.49 pg/mL, range: 2.65–12.50 pg/mL, 44%) and 54 male (4.98 pg/mL, range: 1.84–25.00 pg/mL; 56%) indicated no significant sex difference (95% CI [–0.510, 1.100], *P* = 0.486; Fig. 1C).

### Serum and CSF NfL levels in treatment‐naïve SMA patients

**Figure 2:**
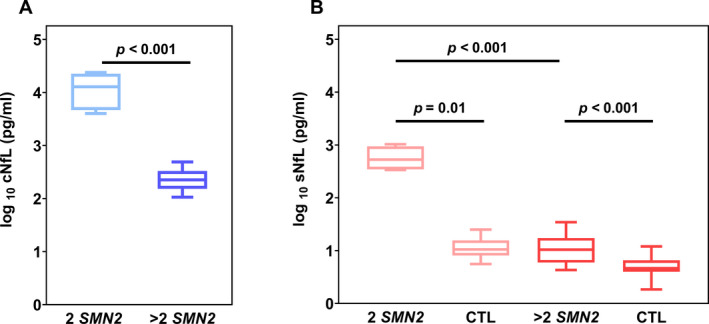
Neurofilament concentrations in patients with SMA and age‐matched controls. NfL values of patients with 2 *SMN2* copies and patients with >2 *SMN2* copies (A) in CSF (cNfL, shades of blue) (B) and serum (sNFL, shades of red) compared to their age‐matched neurologically healthy controls (CTL). Boxplots show minimum, maximum, and median levels as well as the interquartile range (25% and 75% percentile). Significant differences between groups are displayed by *P* values (Mann–Whitney‐U test).

The population of 18 SMA patients consisted of 10 females (56%) and 8 males (44%), comprising 4 children (22%) with infantile‐onset SMA (2 *SMN2* copies, most likely classified as SMA I) with a median age of 2.8 months (age range: 18 days–11.1 months) and 14 children (78%) with later‐onset SMA (>2 *SMN2* copies, most likely classified as SMA II–III) with a median age of 8.4 years (age range: 1.1–17.2 years; Table 1). Baseline NfL values in both body fluids, CSF and serum, of the 18 SMA patients before the initiation of nusinersen treatment (first dose, month 0) were significantly higher within the group of patients with 2 *SMN2* copies than in those with >2 *SMN2* copies (95% CI; CSF [3855, 23541], serum [327, 1019], *P* < 0.001; Fig. 2A and B). In CSF the NfL medians were 12795 pg/mL (range: 4020–23800 pg/mL, *n* = 4) and 226 pg/mL (range: 106–489 pg/mL, *n* = 14) for patients with 2 *SMN2* copies and patients with >2 *SMN2* copies, respectively. In serum, the NfL median of patients with 2 *SMN2* copies aged <1 year were 529 pg/mL (range: 335–1030 pg/mL, *n* = 4) and thus 50‐fold increased (95% CI [323, 1021], *P* = 0.010) in comparison to the sNfL median of their age‐matched controls (10.53 pg/mL, range: 5.57–25.00 pg/mL, *n* = 6). SMA patients with >2 *SMN2* copies aged between 1 and 17 years exhibited a median sNfL level of 10.42 pg/mL (range: 4.29–34.50 pg/mL, *n* = 13), which is two times higher (95% CI [1.990, 6.880], *P* < 0.001) than the median of their age‐matched controls (4.65 pg/mL, range: 1.84–12.00 pg/mL, *n* = 52).

### Changes in serum and CSF NfL levels in nusinersen‐treated SMA patients over time

**Figure 3:**
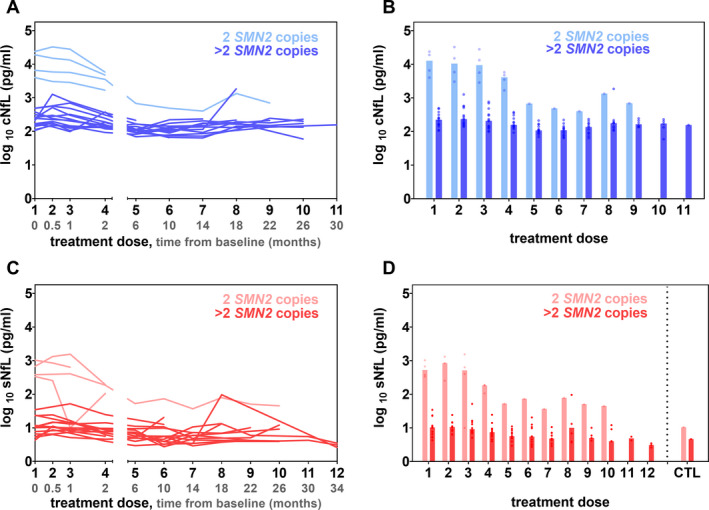
(A, C) cNfL (shades of blue) and sNfL (shades of red) concentrations in pg/mL over time before and during treatment with nusinersen for individual participants (*n* = 18). The two subgroups of SMA patients with 2 *SMN2* and >2 *SMN2* copies are displayed in different color intensities. The *x*‐axis shows treatment doses (black) and time from the initiation of nusinersen treatment (baseline, grey) in months. (B) Median cNfL levels and (D) median sNfL values represented by bars with dots showing individual values in comparison to the median sNfL values of healthy age‐matched controls (CTL).

In patients with 2 *SMN2* copies, NfL levels monotonically decline, most notably observed between treatment dose 1 through 7 in cNfL (Fig. 3A ans B) and treatment dose 2 through 5 in sNfL (Fig. 3C and D), with a relative plateau thereafter. An initial sNfL concentration increase after two weeks of treatment remains statistically insignificant (*P* > 0.999). Within the course of time, sNfL concentrations approach the aforementioned age‐matched median control value of 10.53 pg/mL, with the lowest median sNfL level of 36.80 pg/mL remaining approximately three times higher. In contrast, in the 14 SMA patients with >2 *SMN2* copies, sNfL values are generally close to those of their age‐matched controls (4.65 pg/mL) and there is no apparent downward trend (Fig. 3C, D). Median NfL changes over time for both patient groups are summarized in Table 2.

**Table 2:** Course of NfL levels in nusinersen‐treated SMA patients.

Dose	Time after treatment initiation (months)	2 *SMN2* copies		>2 *SMN2* copies	
		sNfL (pg/mL)	cNfL (pg/mL)	sNfL (pg/mL)	cNfL (pg/mL)
1	0	529 (335–1030)	12795 (4020–23800)	10.42 (4.29–34.50)	226 (106–489)
2	0.5	848 (254–1320)	10485 (3050–32600)	10.60 (6.31–24.30)	234 (138–1270)
3	1	519 (10–1550)	9485 (2870–28000)	9.23 (5.11–52.00)	209 (98–767)
4	2	185 (106–191)	4130 (1680–5790)	7.47 (4.00–24.70)	157 (93–376)
5	6	52.60	677	5.68 (2.94–11.10)	107 (69–213)
6	10	73.10	488	5.51 (2.74–20.50)	109 (65–221)
7	14	36.80	403	4.81 (2.90–10.60)	136 (63–234)
8	18	78.90	1340	10.04 (3.72–96.40)	177 (107–1860)
9	22	50.70	698	5.04 (4.19–10.10)	165 (134–246)
10	26	45.00		4.04 (3.94–12.10)	172 (59–230)
11	30			4.88 (4.26–5.50)	156
12	34			3.07 (2.59–3.48)	

Median (+ range) sNfL and cNfL levels (pg/mL) of SMA patients with 2 *SMN2* and >2 *SMN2* copies under treatment with nusinersen (referring to number of nusinersen dose administrations and corresponding time from baseline in months).

### Correlation of serum and CSF NfL concentrations and association with motor performance

**Figure 4:**
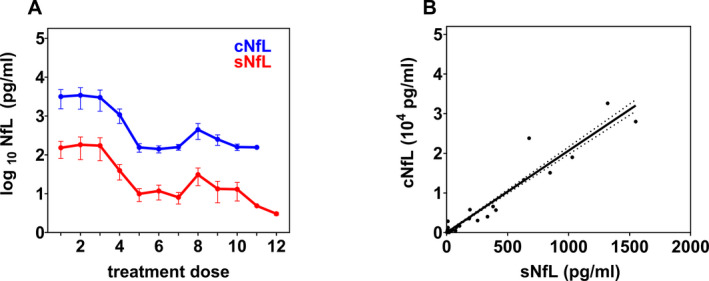
(A) Neurofilament concentrations in CSF (cNfL) and serum (sNfL) of all SMA patients during the course of nusinersen treatment (corresponding time points see Fig. 3A). Data points and error bars correspond to means and SEM, respectively. (B) Correlation between all cNfL and sNfL values of all SMA patients. The linear regression line and 95% confidence interval (dotted curves) are depicted

**Figure 5:**
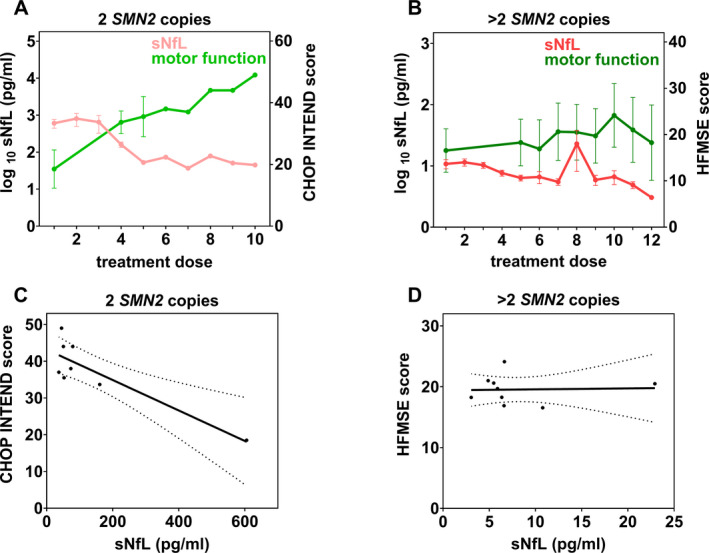
(A, B) Mean (± SEM) sNfL levels (left *y*‐axis) and motor function scores (right *y*‐axis) over time during therapy with nusinersen (at the corresponding treatment dose administrations) of SMA patients with 2 *SMN2* copies measured by CHOP INTEND (A) and SMA patients with >2 *SMN2* copies measured by HFMSE (B). (C, D) Correlation between sNfL levels and motor function scores of SMA patients with 2 *SMN2* copies (C) and >2 *SMN2* copies (D). Data points represent the means of sNfL values and motor scores from panels A and B of time points, in which both measurements were simultaneously determined. The linear regression line and the 95% confidence interval (dotted curves) are depicted.

To assess whether NfL concentrations in CSF and serum are correlated, we calculated the Spearman and Pearson correlation coefficient (*r*) for 120 paired CSF and serum samples of the 18 SMA patients. A high correlation between NfL in both compartments was observed (Spearman: 95% CI [0.575, 0.773], *r* = 0.7, *P* < 0.001; Pearson: 95% CI [0.955, 0.978], *r* = 1.0, *P* < 0.001; Fig. 4; correlation of SMA patient subgroups see Supplementary Figure S1). Due to this strong correlation, sNfL values were used to further evaluate associations between changes in NfL and motor function. Depending on the patient's age, motor function during the course of nusinersen treatment was evaluated either by CHOP INTEND (<2 years) or by HFMSE scales (>2 years). Therefore, with the exception of patient #5, individuals assessed by CHOP INTEND reflect the motoric development of SMA patients with 2 *SMN2* copies. Children assessed by the HFMSE constitute the cohort of SMA patients with >2 *SMN2* copies and later‐onset disease. Although presenting >2 *SMN2* copies, the motor skills of patient #5 were age‐appropriately measured using the CHOP INTEND and analyzed individually. At baseline, prior to treatment initiation, SMA patients with 2 *SMN2* copies scored the CHOP INTEND with 18.5 ± 6.2 points (mean ± SEM, *n* = 4). Administration of nusinersen was associated with both, a steady improvement in motor function and a decline of NfL concentrations in serum (Fig. 5A). The baseline score of SMA patients with >2 *SMN2* copies evaluated by the HFMSE was 16.5 ± 4.7 points (mean ± SEM, *n* = 11). No relevant changes in motor function or NfL concentration were observed during the period of nusinersen treatment (Fig. 5B). These results suggest that an increase in motor function and a decrease in NfL concentrations are negatively correlated in SMA patients with 2 *SMN2* copies and uncorrelated in SMA patients with >2 *SMN2* copies. The Spearman and Pearson correlation coefficients confirmed a moderate to strong negative correlation between the increase in CHOP INTEND scores and decrease of sNfL concentrations (Spearman: *r* = –0.6, *P* = 0.134; Pearson: 95% CI [–0.975, –0.414], *r* = –0.9, *P* = 0.005, Fig. 5C). In contrast, no correlation was observed between HFMSE scores and the corresponding sNfL values (Spearman: *r* = –0.1, *P* = 0.744; Pearson: 95% CI [–0.642, 0.686], *r* = 0.0, *P* = 0.920, Fig. 5D).

In view of the previous lack of data concerning NfL concentrations in younger patients with later‐onset SMA at initial stages of the disease during early childhood, patient #5, the youngest SMA patient of our cohort with >2 *SMN2* copies, was analyzed more closely. Due to his age of 1 year at the time of treatment initiation, he was the only patient with >2 *SMN2* copies to be tested with the CHOP INTEND. Starting from a baseline score of 32 points, an increase in motor function with a maximum of 44 points at treatment dose 7 (14 months after the initiation of treatment) was noted and the Spearman and Pearson correlation coefficients revealed a strong inverse correlation with the changes in the patient’s sNfL levels during therapy (Spearman: *r = *–1.0, *P* = 0.333; Pearson: *r* = –0.9, *P* = 0.236).

## Discussion

The increasing availability of therapeutic options for patients with SMA on one hand and heterogeneous clinical response to therapies on the other hand, emphasizes the need for reliable biomarkers to monitor disease activity and treatment response. Results of previous studies that explored both, neurofilament light chain (NfL) and neurofilament heavy chain (NfH), as blood biomarkers in different types of SMA are inconclusive, depending on the subtype.^9^, ^11^ Both proteins are exclusively expressed in the cytoskeleton of neurons, yet differ in their molecular weight, stoichiometric distribution, and posttranslational modifications, which affect their release and clearance dynamics.^12^, ^18^ To assess the value of NfL as a diagnostic and monitoring biomarker in pediatric SMA patients of different ages, we investigated whether NfL values in serum and CSF reflect changes of disease activity and treatment response in children and adolescents with SMA and different *SMN2* copies over an extended period of up to 34 months. Using ultrasensitive SiMoA immunoassays, we additionally investigated serum NfL in a large group of healthy controls to establish reference data across the pediatric age‐span. Owing to its analytical quality and inter‐laboratory reproducibility,^19^ the presented control cohort may be instrumental as NfL‐reference range for further studies of pediatric neurodegenerative disorders. Similar to previous data in adults, our results in the pediatric control cohort revealed no gender differences in sNfL concentrations.^20^ We identified a bimodal age distribution of two significant intervals that discriminates children between 0 and 4 years of life and older children/ adolescents between 5 and 18 years. The higher NfL concentrations observed in the younger age group are in agreement with pediatric control values obtained by other colleagues^21^ and may reflect the physiological neuronal programmed cell death as part of neuronal network reconfigurations within the developing CNS.^22^ Likewise, immature clearance mechanisms may also contribute to higher NfL concentrations.^23^

Serum NfL values in untreated SMA patients were significantly higher than their age‐matched controls and associated with severity of disease. sNfL concentrations in children with SMA and 2 *SMN2* copies were more than 50‐fold higher than in individuals with later‐onset SMA and >2 *SMN2* copies. A more precise distinction between individuals with 3 and 4 *SMN2* copies was not feasible considering an unequal sample size to the disadvantage of individuals with 4 *SMN2* copies. During our observation period of up to 34 months, cNfL and sNfL levels in children with SMA and 2 *SMN2* copies decrease while those in children with SMA and >2 *SMN2* copies only marginally decrease during the course of treatment. This is not unexpected, since their NfL values are (although statistically significant) only moderately different from values of neurologically healthy children and further converge toward sNfL levels of controls within the observed treatment period. Analogously, their cNfL values, prior to the initiation of treatment, are within a previously reported reference interval of adults measured by ELISA (<380 pg/mL; UmanDiagnostics, Umea, Sweden).^24^ The highly elevated pre‐treatment NfL concentrations seen in infantile‐onset SMA are in line with previously reported levels of cNfL and NfH in plasma of treatment‐naïve SMA patients with 2 *SMN2* copies.^9^, ^10^ These data help to understand the natural history of disease that is characterized by a rapid decline of functional abilities during the first 6 months of life.^25^ Besides, higher pre‐treatment NfL concentrations were not only associated with disease subtype but also younger age (cNfL vs. age: Spearman: 95% CI [–0.871, –0.286], *r* = –0.7, *P* = 0.002; Pearson: 95% CI [–0.798, –0.082], *r* = –0.5, *P* = 0.024; sNfL vs. age: Spearman: 95% CI [–0.944, –0.598], *r* = –0.8, *P* < 0.001; Pearson: 95% CI [–0.838, –0.166], *r* = –0.6, *P* = 0.011). The age association is highlighted by patient #5, the youngest SMA patient with 3 *SMN2* copies, who at the age of 1 year with 34.50 pg/mL exhibited a considerably higher initial concentration than the other SMA patients with >2 *SMN2* copies (4.29–23.60 pg/mL). These data are particularly valuable as they fill an information gap of NfL values in patients with later‐onset SMA within their initial stages of disease during early childhood, as previously pointed out by Wurster et al.^11^

We detected one outlier of one patient carrying >2 *SMN2* copies at the 8^th^ nusinersen administration that was notable both, in CSF and serum. Taking common standard conditions and a high degree of pre‐analytical stability into account,^26^ the simultaneous NfL increase in both compartments suggests a previous intra‐individual neuronal injury, for example, as part of a disease flare‐up or infection. A recent experimental study in macaque monkeys suggests that repeated CSF sampling by lumbar puncture can lead to iatrogenic NfL elevations, likely caused by nerve root damage of the cauda equine.^27^ As long as comprehensive kinetic studies are still pending, this possible confounder should be taken into consideration, especially in those SMA patients in whom progressive neuromyopathic scoliosis complicates lumbar punctures. A less invasive route of sampling, particularly in childhood, that is collection of blood, is desirable for an ideal biomarker of neuronal damage. In this regard, our evaluation of the relationship between CSF‐ and serum‐NfL pairs revealed a high positive association. Analogous to findings in adults,^28^ this result indicates, that measurements of NfL in the peripheral blood of children reflect neuroaxonal CNS damage as reliable as their measurements in CSF. A study by Wurster et al. described a positive correlation of CSF and serum NfL levels in adult patients with SMA II but not with SMA III.^11^ We determined a positive correlation in both subgroups of our pediatric patient cohort.

So far, changes in SMA disease status and treatment response are described by clinical alterations in motor function. While significant clinical improvements are the best evidence of efficacy and should be the primary outcome measure, in the light of a well‐recognized inter‐ and intraindividual variability in motor development, additional reliable molecular biomarkers that objectively assert disease states and therapeutic responses of children with SMA would expand current possibilities.

In agreement with previous studies, we found that decreasing sNfL concentrations in SMA patients with 2 *SMN2* copies, who were younger than 1 year at the initiation of nusinersen treatment, were associated with an increase in motor performance evaluated by the CHOP INTEND.^9^, ^10^ In contrast, the mild improvement of motor function of the older individuals with SMA and >2 *SMN2* copies, evaluated by the HFMSE, did not correlate with sNfL changes, confirming data by Wurster et al. from a shorter observational period.^11^ As only exception, the considerably younger patient #5 with 3 *SMN2* copies showed substantial improvements in motor skills, which were closely correlated with changes in sNfL concentrations during treatment (Spearman: *r = *–1.0, *P* = 0.333; Pearson: *r* = –0.9, *P* = 0.236). We, therefore, hypothesize whether the age at the initiation of treatment is an even more decisive determinant of future changes in NfL and motor function achievements than the *SMN2* copy number.

The physical abilities during the course of disease in older patients (>3 years) with SMA and >2 *SMN2* copies lack correlation with NfL values. Their NfL levels were comparatively lower than those of SMA patients with 2 *SMN2* copies and close to control values, suggesting no or only marginal neuroaxonal degeneration. These observed low values close to controls, even prior to the initiation of treatment, are in agreement with NfL levels observed in adult patients with SMA and >2 *SMN2* copies.^11^ They could be explained by a very slow natural disease progress or could indicate that in those patients the natural disease course may not be dominated by motor neuron loss, but rather other non‐neurodegenerative mechanisms, such as glia‐mediated neuroinflammation or muscular pathologies.

Evidence for a mutual influence between motor neurons and glial cells as well as glial‐mediated immune activation come from different experimental cellular and animal models, recently reviewed by Abati et al.^29^ Correspondingly, gliosis and glial bundle formation have been observed in post‐mortem spinal cord and brain stem of SMA patients.^30^, ^31^

Notably, in an SMA mouse model, the muscle‐specific SMN reduction has been described to transition to a cell‐autonomous muscle pathology as a consequence of ongoing low SMN levels.^32^ Chronic activity‐related serum creatine kinase elevations observed in SMA patients, persisting despite nusinersen‐treatment, presumably reflect this chronic continuing muscle disorder. Other experiments, reviewed by Groen et al., revealed an important temporal regulation of SMN expression at the neuromuscular junction during development^33^ with highest protein levels in the human spinal cord during early embryogenesis and decline within the first three postnatal months, possibly indicating a particular need for SMN protein during this developmental stage.^34^ Earlier diagnosis and initiation of treatment could prevent motor neuron loss during this postnatal vulnerable period. Consistent with this notion, newborn screening (NBS) programs for SMA are explored and implemented in a growing number of countries.^35^, ^36^ Whether sNfL may serve as a biomarker of disease activity, able to confirm the urgency of therapy in presymptomatic children, needs to be further studied. Recently, Otsuki et al. established a promising semi‐quantitative analysis, on the basis of which SMN protein changes in a peripheral blood nuclear cell population may be monitored using imaging flow cytometry.^37^ As such, NfL and SMN protein are potential complementary SMA biomarker candidates that could reflect disease activity and therapeutic efficacy out of different angels, with SMN protein count as specific positive verification of efficient therapeutic upregulation and NfL as a negative indicator of ongoing neuroaxonal injury. Our data on NfL in SMA patients, along with findings of other groups, raise questions about whether low treatment‐naïve NfL concentrations within reference ranges may indicate a missed window of opportunity for SMN modifying interventions.

Taken together, our broad pediatric control cohort may serve as reference values for further studies investigating sNfL in pediatric neurodegenerative diseases. Given the rarity and heterogeneity of SMA, a limitation of the study is the relatively small number of patients. While further research is required to validate these findings, we conclude that serum NfL values in children and adolescents with SMA and varying *SMN2* copies reliably reflect CSF values, regardless of the underlying subtype. Serum NfL concentrations are correlated with clinical changes in motor function in SMA patients with 2 *SMN2* copies and one SMA patient with >2 *SMN2* copies within his first 2 years of life. With regard to the availability of different disease‐modifying therapies, monitoring of sNfL in these children may serve as a complementary early indicator of treatment response and help to stratify disease management. In patients with >2 *SMN2* copies who are older than 3 years, sNfL values differ only moderately from age‐matched controls and fail to mirror changes in motor function, suggesting that in later stages of SMA the pathophysiological process of neurodegeneration may be marginal or play a minor role than previously anticipated. This hypothesis, together with the question of whether sNfL elevations could delineate a critical window of susceptibility for effective SMN‐modifying interventions, encourage further investigation.

## Author Contributions

Study concept and design: M.v.d.H. and V.T. Acquisition, analysis and interpretation of data: all authors. Drafting of the manuscript: E.N., M.v.d.H. and V.T. All authors reviewed and approved the final version of the manuscript.

## Conflict of Interest

M.S. and M.v.d.H. participated in advisory boards and received compensations for presentations by Avexis/Novartis, Biogen, and Roche. K.A. has received personal compensation for consulting services from Alexia, Teva, Roche, Celgene, and Sanofi that are all outside the submitted work. T.Z. reports grants and personal fees from Bayer, grants, and personal fees from Biogen, grants and personal fees from Teva, grants and personal fees from Genzyme, grants and personal fees from Novartis, personal fees from Merck, personal fees from Almirall, personal fees from Roche that are all outside the submitted work.

## Supporting information

Supplementary Figure S1. Correlation between all available cNfL and sNfL values of SMA patient subgroups with (A) 2 *SMN2* copies (Spearman: 95% CI [0.823, 0.975], *r* = 0.9, *P* < 0.001; Pearson: 95% CI [0.867, 0.980], *r* = 0.9, *P* < 0.001) and (B) >2 *SMN2* copies (Spearman: 95% CI [0.325, 0.632], *r* = 0.5, *P* < 0.001; Pearson: 95% CI [0.662, 0.831], *r* = 0.8, *P* < 0.001) showing the linear regression line and the 95% confidence interval (dotted curves).

Supplementary Table S1. Number of patient samples at different time points and dose administrations. Number of cNfL and sNfL samples at each time of measurement (with the corresponding number of nusinersen dose administration) in total and respective SMA subtypes (patients with 2 *SMN2* copies and patients with >2 *SMN2* copies).
