# Supplementary figures and images for: Serum neurofilament light chain in pediatric spinal muscular atrophy patients and healthy children
Source: Ann Clin Transl Neurol. 2021 Sep 4;8(10):2013–24. doi: 10.1002/acn3.51449 (PMC8528467; doi:10.1002/acn3.51449)

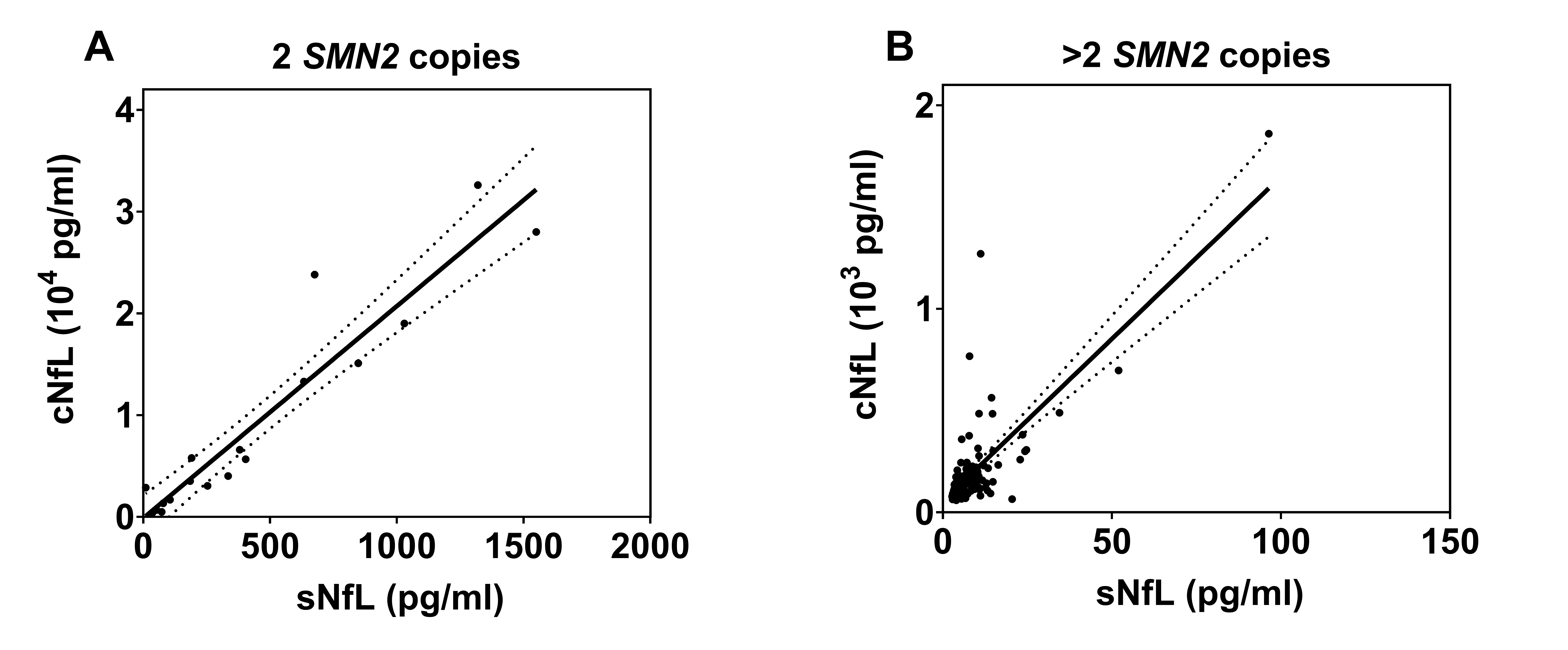

Supplement: Supplementary file 1 — Supplementary Figure S1. Correlation between all available cNfL and sNfL values of SMA patient subgroups with (A) 2 SMN2 copies (Spearman: 95% CI [0.823, 0.975], r = 0.9, P < 0.001; Pearson: 95% CI [0.867, 0.980], r = 0.9, P < 0.001) and (B) >2 SMN2 copies (Spearman: 95% CI [0.325, 0.632], r = 0.5, P < 0.001; Pearson: 95% CI [0.662, 0.831], r = 0.8, P < 0.001) showing the linear regression line and the 95% confidence interval (dotted curves). [file ACN3-8-2013-s001.tif]
