# Supplementary material for: Serum neurofilament light chain in pediatric spinal muscular atrophy patients and healthy children
Source: Ann Clin Transl Neurol. 2021 Sep 4;8(10):2013–24. doi: 10.1002/acn3.51449 (PMC8528467; doi:10.1002/acn3.51449)
Supplement: Supplementary file 2 — Supplementary Table S1. Number of patient samples at different time points and dose administrations. Number of cNfL and sNfL samples at each time of measurement (with the corresponding number of nusinersen dose administration) in total and respective SMA subtypes (patients with 2 SMN2 copies and patients with >2 SMN2 copies). [file ACN3-8-2013-s002.docx]

**Supplementary Table 1.** Number of patient samples at different time points and dose administrations

|  |  | **Number of**  **cNfL samples** | | | **Number of**  **sNfL samples** | | |
| --- | --- | --- | --- | --- | --- | --- | --- |
| **Dose** | **Time after treatment initiation (months)** | **Total** | **2**  ***SMN2*** | **>2 *SMN2*** | **Total** | **2**  ***SMN2*** | **>2 *SMN2*** |
| **1** | 0 | 18 | 4 | 14 | 17 | 4 | 13 |
| **2** | 0.5 | 18 | 4 | 14 | 14 | 3 | 11 |
| **3** | 1 | 18 | 4 | 14 | 16 | 4 | 12 |
| **4** | 2 | 17 | 4 | 13 | 15 | 3 | 12 |
| **5** | 6 | 15 | 1 | 14 | 13 | 1 | 12 |
| **6** | 10 | 14 | 1 | 13 | 14 | 1 | 13 |
| **7** | 14 | 12 | 1 | 11 | 12 | 1 | 11 |
| **8** | 18 | 10 | 1 | 9 | 7 | 1 | 6 |
| **9** | 22 | 7 | 1 | 6 | 6 | 1 | 5 |
| **10** | 26 | 5 |  | 5 | 6 | 1 | 5 |
| **11** | 30 | 1 |  | 1 | 2 |  | 2 |
| **12** | 34 |  |  |  | 3 |  | 3 |

Number of cNfL and sNfL samples at each time of measurement (with corresponding number of nusinersen dose administration) in total and respective SMA subtypes (patients with 2 *SMN2* copies and patients with >2 *SMN2* copies).
